# MLLT11 siRNA Inhibits the Migration and Promotes the Apoptosis of MDA-MB-231 Breast Cancer Cells

**DOI:** 10.1155/2023/6282654

**Published:** 2023-12-01

**Authors:** Xiangrong Liu, Wenqi Bai, Jianrong Li, Jinfeng Ma, Yan Liu, Zhixiang Wang, Linjie Hu, Zheng Li, Dimitri Papukashvili, Nino Rcheulishvili, Fusheng Wang, Xiaoqing Lu

**Affiliations:** ^1^Shanxi Province Cancer Hospital, Shanxi Hospital Affiliated to Cancer Hospital, Chinese Academy of Medical Sciences, Cancer Hospital Affiliated to Shanxi Medical University, Taiyuan 030001, China; ^2^Breast Surgery, The Second Hospital of Shanxi Medical University, Taiyuan 030001, China; ^3^Shanxi Medical University, Taiyuan 030006, China; ^4^Southern University of Science and Technology, Shenzhen 518055, China

## Abstract

Breast cancer is considered the most prevalent malignancy due to its high incidence rate, recurrence, and metastasis in women that makes it one of the deadliest cancers. The current study aimed to predict the genes associated with the recurrence and metastasis of breast cancer and to validate their effect on MDA-MB-231 cells. Through the bioinformatics analysis, the transcription factor 7 cofactor (MLLT11) as the target gene was obtained. MLLT11-specific siRNA was synthesized and transfected into MDA-MB-231 cells. The results demonstrated that the siRNA significantly reduced the MLLT11 mRNA levels. Moreover, cell migration and invasion, as well as the protein levels of phosphatidylinositol 3-kinase (PI3K), AKT, matrix metalloproteinase (MMP) 2, and MMP9, were significantly lower in the groups treated with siRNA while the apoptosis was augmented. Collectively, MLLT11 siRNA elicited ameliorative properties on breast cancer cells, possibly via the inhibition of the PI3K/AKT signaling pathway.

## 1. Introduction

Despite the recent medical advances, breast cancer remains the most prevalent malignancy and the second in cancer-related deaths in women globally [1]. Currently, breast cancer adopts a comprehensive treatment mode that includes surgery, radiotherapy, chemotherapy, and drug therapy, which improves the survival rate of patients. However, the high mortality rate of women patients is usually caused by metastasis and recurrence that represent the serious obstacles in breast cancer treatment [2, 3]. The genetic diversity of breast cancer induces different clinical features of the cancer cells that hamper the efficient management of cancer. The cancer stem cell (CSC) theory explains the theoretical mechanism for the phenomena of metastasis and recurrence. CSCs are cells with the potential for self-renewal and the ability to develop into cancer cells that drive tumor progression [4]. These characteristics render CSCs resistant to conventional treatment; therefore, controlling CSCs appears to be a critical first step in devising an effective cancer treatment strategy. Thus, CSCs represent a key target in cancer studies and anticancer drug discovery. The standardized chemotherapy regimen implies killing the fast and abnormally proliferating and growing cells. However, CSCs elicit resistance to chemical anticancer drugs. For this reason, the current study employed bioinformatics analysis to predict genes that may be associated with the development, recurrence, and metastasis of breast cancer. This involved studying the intersection of differential gene expression of CSCs in normal reduction mammoplasty and invasive breast cancer patients, which was then diagrammatically represented. Among the predicted genes, the transcription factor 7 cofactor (MLLT11) was selected as a potential target gene for breast cancer therapy. Markedly, small (short)-interfering RNA (siRNA) represents a key molecule for the gene knockdown. It is synthetically obtained small RNA that interferes with gene expression very specifically [5]. Indeed, there are already four siRNAs approved by the Food and Drug Administration (FDA) that are commercially available for the treatment of various diseases [6–9]. Thus, an siRNA specifically targeting MLLT11 was synthesized and experimentally validated in MDA-MB-231 breast cancer cells. The suppression of MLLT11 and the influence on cell migration and invasion were evaluated.

## 2. Materials and Methods

### 2.1. Cell Culture

MDA-MB-231HM cells (Fudan University Shanghai Cancer Center, China) were incubated in DMEM (Gibco, USA) supplemented with 10% FBS and 1% penicillin-streptomycin at 5% CO_2_ and 37°C in a humidified incubator. Normal mammary epithelial MCF10A cells (Manassas, USA) were cultured in a growth medium containing DMEM/F12, 10% horse serum, and 1% penicillin-streptomycin and maintained at 37°C in a humidified atmosphere with 5% CO_2_.

### 2.2. Differential Gene Screening

Datasets GSE6883 and GSE10797 were retrieved from the gene expression database, Gene Expression Omnibus (GEO, https://www.ncbi.nlm.nih.gov/geo/). GSE6883 and GSE10797 were selected using “GEO2R,” and the selection conditions were *P* < 0.05 and |log FC| > 0.5 (GSE6883) and *P* < 0.05 and |log FC| > 1 (GSE10797). The differential genes were divided into total upregulated genes, upregulated genes of GSE6883, and downregulated genes of GSE10797 according to log fold change (FC) values obtained by drawing a Venn diagram. Through the Kaplan–Meier analysis (https://kmplot.com/analysis/), the effects of genes on recurrence-free survival (RFS) in patients with breast cancer were compared.

### 2.3. Cell Transfection

MDA-MB-231 cells were incubated in a culture flask. When the density of cells reached 80%, the cells were digested and passaged. The cells were inoculated in 6-well plates a day before transfection so that during the transfection, the cell density was 50%. The transfection was performed via lipofectamine 2000. MDA-MB-231 cells at the logarithmic growth stage were collected and counted, and the cell density was adjusted to 2-3 *∗* 10^5^ cells/ml. Cells were inoculated in 12-well culture plates, 1 ml per well; Opti-MEM was used to dilute a certain volume of sialic RNA and lipofectamine 2000 (sialic KNf A final concentration 50 nM, lipofectamine 2000 1 : 50), respectively, by MEM, and the plate was kept at room temperature for 5 min. The two suspensions were gently mixed 1 :  1 and kept at room temperature for 20 min to form a complex of sialic RNA and lipofectamine 2000. After 20 min, a small amount of the abovementioned mixture was also added to the cell culture plate and the plate was gently shaken. The plate was then placed in an incubator to allow the MDA-MB-231 cells and the complex to mix well. After 4–6 hours, the medium was replaced. For validating the transfection efficiency, RNA was extracted from the cells after 48 h of incubation.

### 2.4. Real-Time PCR Detection

After RNA extraction from the cells, the cDNA was reversely transcribed and then amplified by PCR. The MLLT11 upstream primer sequence was 5′-GCACTCCCTCCATCTTTGGA-3′ and downstream primer was 5′-CAGCTCCGACAGATCCAGTTC-3′. The GAPDH upstream primer was 5′-CTCTCTGCTCCTCCTCCTGTTCGAC-3′ and downstream primer was 5′-GAAGGAAGGCTGGAAGAGTGTGAGCGATGTGGCTCGGCT-3′. Three independent experiments were performed.

### 2.5. Wound-Healing Assay

The MDA-MB-231 breast cancer cells were seeded in 6-well plates with the density of 1 × 10^6^ cells per well and incubated at 37°C in a 5% CO_2_ incubator under a humid environment until the density reached 100%. Scratch was performed using a 200 *μ*L pipette tip. After washing with PBS, the cells were treated accordingly (experimental groups: blank control group (control group), negative control group (NC group), and RNA-interference group (siRNA group)). Scratches were recorded at 0 h and 48 h using a phase-contrast microscope with an in-built digital camera. The percentage of wound healing = ((scratch area at 0 h − scratch area at 48 h)/(scratch area at 0 h)) × 100. The assay was performed three independent times.

### 2.6. Cell Migration and Invasion Analysis

The chamber was coated with Matrigel in advance and the cells with a density of 40,000 cells per well were seeded in the upper chambers of the 6-well Transwell. The chambers were incubated at 37°C for 48 h in a 5% CO_2_ incubator under humidity. The staining of the lower chamber was performed with crystal violet and photographs were taken.

### 2.7. Annexin V-FITC/PI Staining Assay

MDA-MB-231 cells were seeded in 6-well plates and treated in groups as above. After 48 h, the washing was done by PBS, and cell digestion was performed with trypsin without EDTA. The cells were operated accordingly, and flow cytometry was used for the detection of apoptosis.

### 2.8. Western Blot Analysis

The lysis of cells was performed using radio-immunoprecipitation assay buffer (RIPA) on the ice. The supernatants of the lysate after centrifugation (12,000 g/30 min) were collected, and the proteins were separated by 10% SDS-PAGE and transferred to PVDF membranes. 1 h blocking was done with 5% skimmed milk and the membranes were then incubated with primary antibodies anti-STAT3, anti-STAT3 (phospho Y705), and anti-*β*-actin (Abcam, Cambridge, UK) (1 : 2000) at 4°C overnight. The membranes were incubated with the secondary antibodies (1 : 1000) for 2 h at room temperature. After incubation with the first and second antibodies, the membranes were washed three times using TBST. The images were obtained by an automatic gel imaging system.

### 2.9. Statistical Analysis

Statistical software SPSS 19.0 was employed for data analysis. The data are presented as the mean ± standard deviation of the mean of the independent experiments. The *t*-test was used for comparison between the two groups, and ANOVA was employed for determination of differences among the groups. *P* values that were less than 0.05 (*P* < 0.05) were considered to indicate a significant difference.

### 2.10. Animal Experiments

Fifteen 6-week-old BALB/C female mice were divided into three groups as follows: the control group, siRNA group, and inhibitor group. MDA-MB-231 cells were diluted to 1 × 10^7^ cells/ml with PBS, and 100 *μ*l of cell suspension (1 × 10^6^ cells) was injected into the abdominal mammary fat pad of 6-week-old BALB/C female mice. The control group, siRNA group, and inhibitor group were injected with PBS solution, MLLT11 siRNA, and inhibitors (activators of CMA, including 6-aminonicotinamide and nutrient starvation) after tumor formation, respectively. The tumor size was observed after four weeks.

## 3. Results

### 3.1. Differential Gene Screening

**Figure 1:**
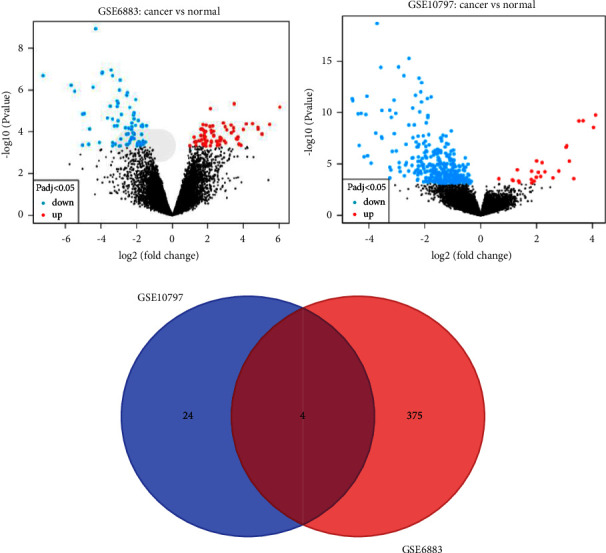
Differential gene screening. (a) Volcano plot of differentially expressed genes (DEGs) in the GSE6883 dataset. Volcano map of DEGs: DEG is represented by each colored dot based on the criteria of *P*  <  0.05 and |log FC| > 0.5. (b) Volcano plot of DEGs in the GSE10797 dataset. Volcano map of DEGs: DEG is represented by each colored dot based on the criteria of *P*  <  0.05 and |log FC| > 1; red: upregulation; blue: downregulation; gray: not significantly expressed genes. (c) Venn diagram of the abovementioned two datasets.

After selection, 379 upregulated and 340 downregulated genes were obtained from the GSE6883 dataset (Figure 1(a)), while 28 upregulated genes and 2114 downregulated genes were obtained from the GSE10797 dataset (Figure 1(b)). Four intersected genes (Figure 1(c)) were found by the intersection of the upregulated genes of GSE6883 and upregulated genes of GSE10797, which were as follows: GPRC5A, MLLT11, RRM2, and NAT1.

### 3.2. Relationship between the Expression of Differential Genes and Prognosis

**Figure 2:**
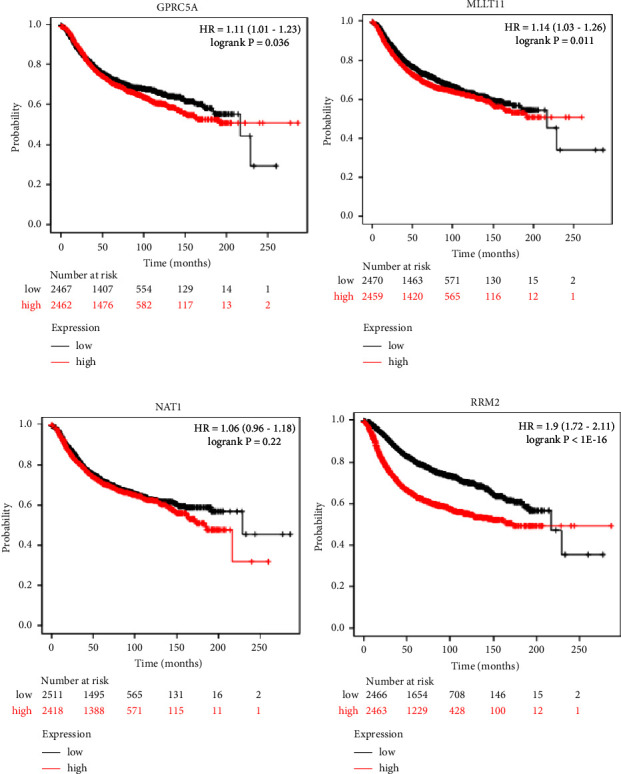
Genes associated with the survival outcomes of patients. (a) Recurrence-free survival outcome associated with GPRC5A gene expression. (b) Recurrence-free survival outcome associated with MLLT11 gene expression. (c) Recurrence-free survival outcome associated with NAT1 gene expression. (d) Recurrence-free survival outcome associated with RRM2 gene expression. High gene expression is represented with the red lines, and low gene expression is represented with the black lines.

Four key genes related to prognosis were obtained after survival analysis of the differential genes, namely, GPRC5A, MLLT11, RRM2, and NAT1 (all *P* values less than 0.05). GPRC5A, MLLT11, RRM2, and NAT1 are related to RFS (Figures 2(a)–2(d)). Combining the RFS maps of the four genes, GPRC5A and MLLT11 should be selected for exploration. Based on the fact that GPRC5A is less studied and lacks its specific association with cancer, we chose MLLT11 for a deeper study.

### 3.3. MLLT11 mRNA Level in MDA-MB-231 and MCF10A Cells

**Figure 3:**
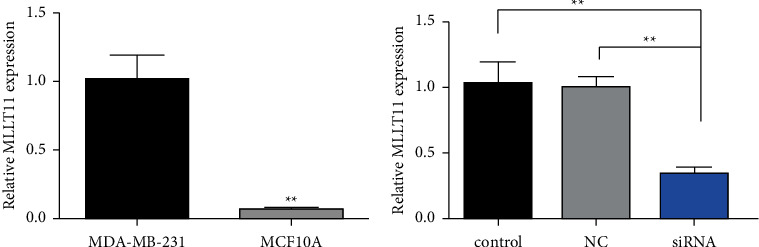
Relative concentration of MLLT11 mRNA and gene expression interference efficiency. (a) Relative mRNA expression of MLLT11 in MDA-MB-231 and MCF10A cells. (b) Interference efficiency of MLLT11-specific siRNA. ^*∗∗*^*P* < 0.01.

The level of MLLT11 in MDA-MB-231 cells was significantly higher when compared with that in the normal mammary epithelial MCF10A cells (*P* < 0.01, Table 1); MLLT11 was highly expressed in MDA-MB-231 cells (Figure 3(a)). Therefore, MDA-MB-231 cells were selected for the knockdown test. The siRNA interference with MLLT11 gene expression analysis showed that the MLLT11 levels in the siRNA group were significantly lower than in the control and NC groups (*F* = 12.387; *P* < 0.01, Figure 3(b)). The gene expression of each group is shown in Table 2.

### 3.4. Migration and Invasion of MDA-MB-231 Cells

**Figure 4:**
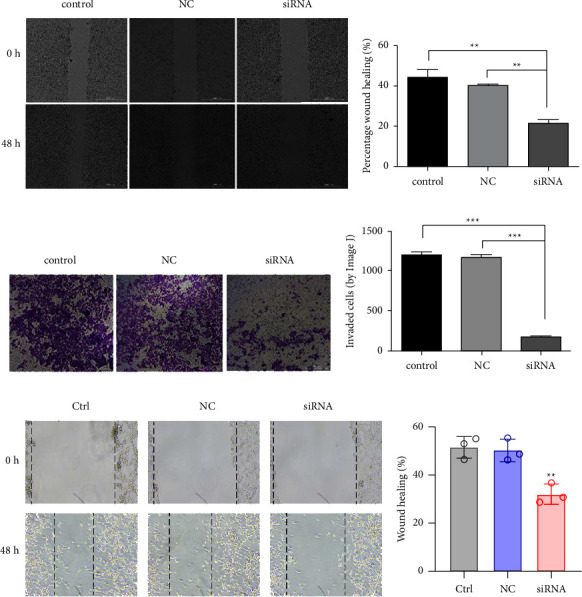
Migration and invasion of MDA-MB-231 cells. (a) Representative images from scratch wound healing of MDA-MB-231 cells, assays acquired at 0 and 48 h. (b) Wound-healing ability of MDA-MB-231 cells. (c) Representative images showing the invasion of MDA-MB-231 cells. (d) Invasion ability of MDA-MB-231 cells. (e) Representative images from scratch wound healing of MDA-MB-231 cells; assays acquired at 0 and 48 h with PI3K inhibitor. (f) Wound-healing ability of MDA-MB-231 cells with PI3K inhibitor. ^*∗∗*^*P* < 0.01 and ^*∗∗∗*^*P* < 0.001.

The migration and invasion of breast cancer cells were reduced in the siRNA group. The difference in the wound-healing rate in the control, NC, and siRNA groups was statistically significant by one-way ANOVA (*F* = 11.926; *P* < 0.01, Figure 4(a)). The wound-healing rate of the siRNA group was markedly lower than that of the control group and NC group (*P* < 0.01). The invasion numbers of the control group, NC group, and siRNA group were 1299 ± 34, 1170 ± 36, and 172 ± 4, respectively (obtained by Image J), and one-way ANOVA showed statistically significant differences (*F*  = 468.391; *P* < 0.001, Figure 4(b)). The number of cells in the siRNA group was significantly lower than that in the control group and the NC group (*P* < 0.001). We then added the PI3K inhibitor LY294002 to each of the three groups and observed the healing of the scratch experiment in all three groups. The wound-healing rates in the control and NC groups were still significantly higher than the siRNA group (*P* < 0.01) but lower than the control and NC groups without the addition of PI3K inhibitor (Figures 4(e) and 4(f)).

### 3.5. Apoptosis of MDA-MB-231 Cells

**Figure 5:**
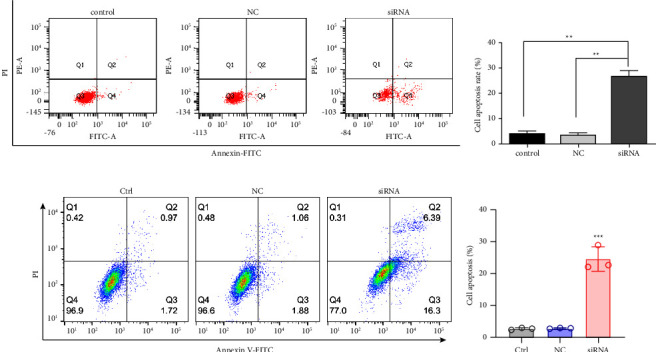
(a) Apoptosis of MDA-MB-231 cells detected by flow cytometry. (b) The apoptosis rate of MDA-MB-231 cells quantified. (c) Apoptosis of MDA-MB-231 cells with PI3K inhibitors detected by flow cytometry. (d) The apoptosis rate of MDA-MB-231 cells with PI3K inhibitors quantified. ^*∗∗*^*P* < 0.01.

Flow cytometry showed that the apoptosis rates of the control, NC, and siRNA groups were (34.47 ± 0.72)%, (36.73 ± 1.10)%, and (47.50 ± 2.17)%, respectively. One-way ANOVA showed that the differences were statistically significant (*F* = 67.903; *P* < 0.001, Figure 5), and the apoptosis rate of the siRNA group was markedly higher compared with the control and NC groups (*P* < 0.001). After the addition of the PI3K inhibitor, the apoptosis rates in the control and NC groups were still smaller than those in the siRNA group but significantly higher than those in the control and NC groups without the addition of inhibitor. These results suggested that the MLLT11 siRNA decreased the proliferation of breast cancer cells through the induction of apoptosis and PI3K and AKT are intermediate signals of MLLT11 affecting tumor development.

### 3.6. Protein Expression in MDA-MB-231 Cells

**Figure 6:**
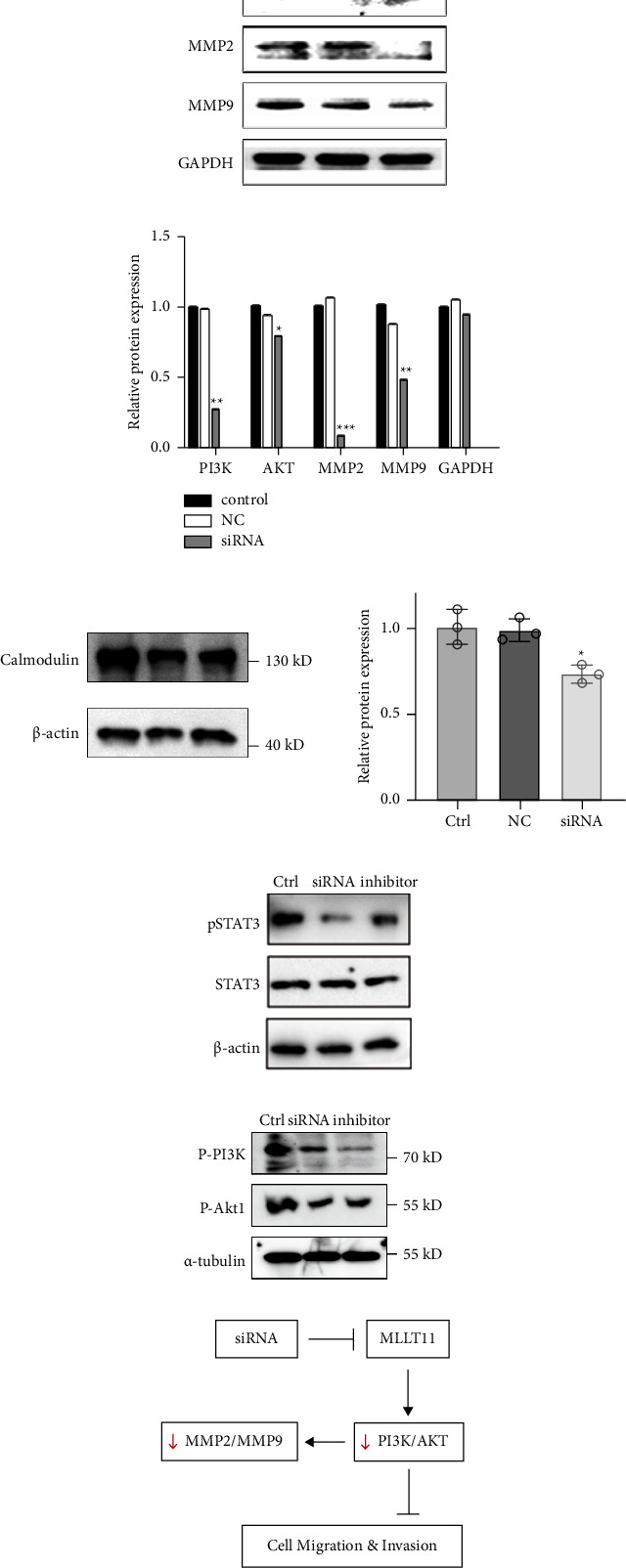
Levels of protein expression in MDA-MB-231 cells. (a) Western blotting of PI3K, AKT, MMP2, MMP9, and GAPDH. (b) Histogram of protein content in each group. (c) Level of calmodulin and relative protein content in each group. (d) Level of pSTAT3 and STAT3 expression in three groups. (e) p-PI3K and p-Akt levels. (f) Schematic illustration of the siRNA effect on MLLT11. ^*∗*^*P* < 0.05, ^*∗∗*^*P* < 0.01, and ^*∗∗∗*^*P* < 0.001.

After transfection of MLLT11 siRNA, MDA-MB-231 cells elicited the downregulation of phosphatidylinositol 3-kinase (PI3K), AKT, matrix metalloproteinase (MMP)2, and MMP9 levels among the three groups (Figure 6). Compared with the control and NC groups, the protein levels of PI3K, AKT, MMP2, and MMP9 in the siRNA-treated group were significantly decreased (*P* < 0.05). Similarly, the biomarker calmodulin was significantly decreased in the siRNA group compared to the control and NC groups. In order to verify the effect of MLLT11 siRNA, the control, siRNA, and inhibitor groups were tested. It was observed that the expression of pSTAT3 was suppressed in the siRNA and inhibitor groups (activators of CMA, including 6-aminonicotinamide and nutrient starvation), thus suggesting a role for the association of MLLT11 with STAT3 in tumor growth (Figure 6(c)).

### 3.7. Effect of MLLT11 on Tumors in Mice

**Figure 7:**
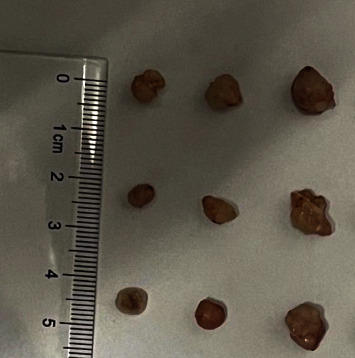
From right to left, the control group, siRNA group, and inhibitor group.

Mice were euthanized and necropsied 4 weeks after cell injection. The tumor size was observed in three groups (Figure 7). It can be concluded that MLLT11 was positively correlated with the mouse mammary tumor size and that the inhibition of MLLT11 resulted in significant tumor shrinkage, consistent with cell line experiments. The schematic illustration showing the effects of siRNA on MLLT11 and its consequence on cell migration and invasion is given in Figure 6(f).

## 4. Discussion

Cancer represents a noncommunicable chronic disease with the rising incidence rate attributed to an aging population as well as lifestyle changes, as reported by the World Health Organization (WHO). Despite numerous attempts to find a cure, until now, cancer remains one of the leading causes of mortality in humans worldwide. Therefore, there is an urgent need to discover an effective cancer treatment. Carcinogenesis comprises the stages of initiation, promotion, progression, and metastasis. Cancer itself is characterized by alterations in cancer cell physiology [10]. Importantly, only a limited number of genes (approximately 140), influencing multiple signaling pathways implicated in cell survival, cell fate, etc., are relevant and denoted as so-called “drivers” of mutations [11]. The PI3K/AKT signaling pathway represents one of the key regulators for cancer that plays a crucial role in proliferation, growth, motility, survival, and angiogenesis in cancer cells [12, 13]. In addition, the levels of proteolytic enzymes MMP2 and MMP9 are also evidenced to contribute to the invasion and metastasis of cancer cells [14], whereas the downregulation of these enzymes together with the inhibition of PI3K/AKT signaling pathway suppresses the migration and invasion of cancer cells in various cancers [14–16], including breast cancer [17].

RNA interference with siRNA has been widely used in studies for silencing gene expression with high specificity [18]. Compared with therapeutic drugs and monoclonal antibodies, siRNAs have a number of advantages that make them quite promising. However, several obstacles such as nonspecific toxicity and off-target effects limit the clinical application of siRNA-based therapy. Moreover, unmodified siRNAs have insufficient stability while circulating in the bloodstream due to the presence of RNses [5]. Nevertheless, siRNAs have advantages such as high potency and high specificity and simplicity of design that make them valuable targets for the treatment of many diseases including cancer [5–9], particularly breast cancer [19].

MLLT11, an oncogenic factor, also known as ALL1-fused gene (AF1q), is located on human chromosome 1q21. It was first identified in patients with acute lymphoblastic leukemia [20]. The present study demonstrated that the MLLT11 expression level is due to the degree of cell differentiation. The expression of MLLT11 was found to be increased in hematopoietic stem cells and hematopoietic progenitor cells. However, its expression decreased with the gradual differentiation and maturation of hematopoietic cells while the expression of MLLT11 in blood cells was low, suggesting that a higher expression level of MLLT11 is associated with the undifferentiated state of stem cells. Elevated MLLT11 expression is also a predictor of poor prognosis [21, 22]. MLLT11 activates the Wnt/*β*-catenin signaling pathway to promote cell proliferation [23] and the Notch signaling pathway to promote cell apoptosis [24] in breast cancer cells. However, there are a limited number of studies on the role of MLLT11 in the metastasis of MDA-MB-231 breast cancer cells. In this study, MLLT11 was predicted as the gene associated with the metastasis and recurrence of breast cancer. The expression level of MLLT11 in normal breast epithelial cells MCF10A and MDA-MB-231 was detected, and the results showed that the expression level of MLLT11 in breast cancer epithelial cells was markedly higher than that in normal breast epithelial cells. Subsequently, MDA-MB-231 breast cancer cells were selected as research objects. For the assessment of the effects of MLLT11 gene knockdown, MDA-MB-231 cells were treated with MLLT11-specific siRNA. As a result, siRNA induced a significant reduction of MLLT11 mRNA compared with the control groups. The results obtained from experiments conducted on cell lines also suggest that MLLT11 may have a positive correlation with the size of mammary tumors in mice. Furthermore, inhibiting MLLT11 led to a significant decrease in the tumor size, which is in line with the outcomes observed in the cell line experiments. In addition, the wound-healing rate and the number of invasive cells in the knock-down group were also markedly reduced compared with the control groups, suggesting that MLLT11 may play an important role in the inhibition of the migration and invasion of MDA-MB-231 cells. Furthermore, the apoptosis rate in the siRNA group was significantly higher compared with the control groups. Consequently, protein expression levels of PI3K, AKT, MMP2, and MMP9 that are the proteins implicated in cancer progression in the siRNA-treated group were significantly decreased, stipulating that the cancer-promoting effect of MLLT11 might be related to the PI3K/AKT signaling pathway. After MLLT11 knockdown, the invasion ability of MDA-MB-231 breast cancer cells was lowered which might be associated with the decreased levels of MMP2 and MMP9 proteins. Besides, the concentration of calmodulin, which serves as a crucial mediator of cellular processes, and its elevated level contributes to their aggressive behavior–increasing tumor invasion and migration, was also decreased in the siRNA group. It was also observed that the expression of pSTAT3 was diminished in the groups treated with both the siRNA and the inhibitor, which are known to activate CMA through 6-aminonicotinamide and nutrient starvation. These results suggest that the association of MLLT11 with STAT3 may play a role in tumor growth. Along with the findings, some limitations in this study, e.g., the sample size of the GEO dataset needs to be mentioned. Besides, verification of the positive impact of MLLT11 knockdown with siRNA in an actual clinical sample is needed. However, the results of this study provide valuable insights into the mechanism of breast cancer recurrence and metastasis, thereby offering a framework for the development of target inhibitors to address this critical issue. In the current era of big data, it is imperative to transition from single biological research to a multidisciplinary approach that leverages bioinformatics. Mining key data and integrating knowledge across various fields allow us to more comprehensively discuss the molecular mechanisms of diseases from various aspects and explore new ideas for treatment strategies.

## 5. Conclusion

In summary, the results of the current study showed that MLLT11 may play an important role in the migration of MDA-MB-231 breast cancer cells and its knockdown may promote apoptosis. Detection of the MLLT11 expression level not only contributes to the prognosis of breast cancer patients but also serves as an important basis for subsequent treatment. The increased expression of MLLT11 in breast cancer patients allows timely prediction of treatment response. Monitoring the expression level of this protein allows for the adjustment of the treatment regimen to minimize the risk of recurrence and metastasis after the treatment. Most importantly, siRNA-based therapy aiming at the knockdown of MLLT11 seems to be a promising approach in breast cancer treatment.

## Figures and Tables

**Table 1:** Expression levels of MLLT11 in MCF10A and MDA-MB-231 cells (*x* ± SD).

Cells	MLLT11
MCF10A	0.07 ± 0.01^*∗∗*^
MDA-MB-231	1.03 ± 0.29
*P* value	0.005

^*∗∗*^ *P* < 0.01.

**Table 2:** Comparison of MLLT11 levels in MDA-MB-231 breast cancer cells transfected with siRNA.

Groups	*n*	MLLT11
Control	3	1.03 ± 0.29
NC	3	1.01 ± 0.13
siRNA	3	0.34 ± 0.08^*∗∗*^
*F*	—	12.387
*P* value	—	0.007

^*∗∗*^ *P* < 0.01.

## Data Availability

Datasets GSE6883 and GSE10797 were retrieved from gene expression database, Gene Expression Omnibus (GEO, https://www.ncbi.nlm.nih.gov/geo/). The rest of the data are included in this article; the original data used in this study are available from the corresponding authors upon reasonable request.
